# A critical orientation on muscle marker placement: Multiple angles to consider^☆^

**DOI:** 10.1016/j.jbiomech.2025.113137

**Published:** 2025-12-17

**Authors:** Cameron R. Taylor, Rachel C. Fleming, William H. Clark, Richard L. Marsh, Thomas J. Roberts

**Affiliations:** a Lampe Joint Department of Biomedical Engineering, UNC-Chapel Hill and NC State, Chapel Hill, NC, USA; b Department of Ecology, Evolution, and Organismal Biology, Brown University, Providence, RI, USA

**Keywords:** Muscle geometry, Marker placement, Fascicle length, Measurement accuracy

## Abstract

The muscles that drive animal and human movement are spatially rich in three-dimensional deformation, yet capturing this complexity *in vivo* is extremely challenging. As a result, much of our research relies on a single metric of muscle geometry: fascicle length. A variety of muscle marker technologies, such as sonomicrometry, fluoromicrometry, and magnetomicrometry, have enabled us to monitor muscle fascicle lengths over time. Due to the complex nature of muscle deformation, the three-dimensional placement of these muscle markers is important. We aimed to determine just *how* important that muscle marker placement is. To this end, we simultaneously monitored muscle long-axis distances and fascicle lengths under a variety of muscle actions in an avian gastrocnemius. We found that muscle long-axis distances did not reliably correspond to fascicle lengths, even occasionally showing inverted length changes, and we found that the muscle long-axis distances lacked a consistent relationship with fascicle lengths and even with each other. In summary, we showed that fascicle length cannot in general be predicted from arbitrary muscle tissue length. Our results suggest that improper placement of muscle markers for tracking fascicle lengths cannot be corrected for in post-processing, and we strongly encourage the biomechanist to take special care in placing each muscle marker pair along a fascicle when studying or otherwise monitoring muscle fascicle lengths.

## 1. Introduction

Though humans have been considering musculoskeletal biomechanics for thousands of years, only recently has the scientific community begun to account for the impact of muscle structure on function. Early models of muscle contraction considered muscle contraction as pneumatic (Pearce, 2013), without considering architectural variations. In the 1600′s, several scientists–notably Steno, Swammerdam, and Borelli–independently demonstrated the isovolume, fiber, and pennate characteristics of muscle (Borelli, 1680; Kardel, 1990). These scientists backed their claims with strong scientific evidence, but only in the past hundred years have we begun to fully appreciate the spatial complexity of muscle (Benninghoff and Rollhäuser, 1952; Gans and Bock, 1965; Pfuhl, 1937), and the scientific community now recognizes the rich three-dimensional nature of muscle contraction (Heemskerk et al., 2010; Roberts et al., 2019; Woittiez et al., 1984).

Length and length trajectory of the contractile element (fascicle length and shortening speed) are major determinants of contractile mechanics (Gordon et al., 1966; Hill, 1938). Accordingly, advancements in tools for directly measuring muscle length have primarily focused on assessing fascicle lengths and length changes. Muscle tissue length tracking strategies such as sonomicrometry (Rushmer et al., 1956), fluoromicrometry (Camp et al., 2016), and magnetomicrometry (Taylor et al., 2021), have been applied to this purpose, where “muscle tissue length” is defined as the distance (the length of muscle tissue) between two markers arbitrarily placed apart in a muscle, and does not necessarily refer to fascicle length. All three technologies rely on pairs of markers: piezoelectric transducers for sonomicrometry (speed-of-sound-based measurement), radiopaque beads for fluoromicrometry (x-ray shadow bead localization), and magnetic beads for magnetomicrometry (permanent magnet localization). Each has unique benefits, and likely other muscle marker technologies with further unique characteristics will be developed. Regardless of the strategy used, however, it is imperative that we consider what these measurements tell us, whether they are measuring along the fascicle, along the full length of the muscle, or otherwise.

We wished to explore how important muscle marker placements are in the measurement of muscle fascicle lengths. Recently, we showed that magnetomicrometry can be used to perform untethered muscle tissue length tracking in freely-roaming turkeys (Taylor et al., 2022). In reviewing those results, a curious reader might find it interesting, as we did, that Bird B’s muscle tissue length profile was inverted from that of Birds A and C, despite having markers placed in the same muscle and leg. A similar curiosity can be found in a recently-published paper on non-intuitive aponeurosis observations (Bossuyt et al., 2023), where the distal aponeurosis strains between Sheep 1 and Sheep 4 appear almost negatively correlated, if at all. Such inconsistent results are cause for concern. If our muscle markers do not reliably provide accurate fascicle (or aponeurosis, or other) length measurements, we are in danger of developing incorrect models of how the musculoskeletal system works.

In this work, we explore the extent to which muscle marker data can provide an inaccurate representation of muscle fascicle length changes, even under well-intentioned muscle marker placement (e.g., when placed along a muscle’s long-axis with an unaided attempt to match the pennation angle by varying marker depths). We implant multiple pairs of markers at different depths along the long-axis of gastrocnemius muscles in five turkeys: one pair oriented *at* the pennation angle (which we visually confirm to be inline with a fascicle), one pair in the muscle belly at an angle to the muscle long-axis *below* that of the pennation angle, and one pair in the muscle belly at an angle to the muscle long-axis that has an *opposite sign* to that of the pennation angle. We then compare the resultant signals against one another during a variety of muscle protocols. The results of this work influence our fundamental understanding of muscle mechanics and thus have critical implications in both biomechanics research and biomedical engineering.

## 2. Methods

All animal experiments were approved by the Institutional Animal Care and Use Committee (IACUC) at Brown University. We obtained five adult male eastern wild turkeys (*Meleagris gallopavo silvestris*, body masses 8.15 kg ± 0.75 kg) from local breeders and cared for them in the Animal Care Facility at Brown University on an ad libitum water and poultry feed diet in accordance with institutional guidelines. The turkeys were maintained on anesthesia using 3 to 4 % isoflurane and intubated and actively ventilated during procedure preparations and experiments. We performed all experiments following methods from previous work (Nelson et al., 2004; Stover et al., 2021).

To measure muscle tissue lengths, we inserted three pairs of custom-made 2-mm sonomicrometry transducers. The transducers were routed under the skin from the upper thigh to the exposed lateral gastrocnemius muscle. For implantation, a small incision was made at the surface of the muscle and the transducer inserted using a plastic inserter tube surrounding the lead wires. The inserter tube was removed from the muscle and the lead wires sutured to the surface of the muscle. We implanted one pair of sonomicrometry transducers inline with the fascicles in the proximal gastrocnemius where entire lengths of the fascicles are clearly visible. We then implanted two additional pairs along the muscle’s long axis. Specifically, for each pair, we marked two implantation sites on the superficial surface of the muscle on either side of the muscle’s center, visually confirming that the line between these points was parallel to a line between the proximal and distal tendons. In the first two turkeys, these long-axis implantations were performed while noting but not explicitly focusing on marker depth. In the last three turkeys, we specifically attempted to implant these long-axis pairs with differing orientations relative to the long-axis: one with an angle less than the pennation angle (under-pennate markers), and the other with an opposite-signed angle to that of pennation (reverse-pennate markers). While other two-dimensional orientations are possible, we chose to explore only three of the five shown in Fig. 1A. Sonomicrometry signals were recorded digitally at 411 Hz (Sonometrics TRX Series 8). These recordings were subsequently transferred to the application Igor Pro (Wavemetrics), corrected for outliers and level shifts, and upsampled to 1000 Hz to match the sampling frequency of the servomotor and load cell.

We stimulated muscle contraction via a custom-made nerve cuff, and we controlled muscle lengths and measured muscle forces via a motor and load cell (see Supplementary Methods). See Fig. 2A for details of each muscle protocol.

In the last three turkeys, muscles were then excised and imaged under μCT (see Fig. 1B and Supplementary methods). We note that, due to limitations in our μCT fixation and measurement methodology, Fig. 1B angles do not fully conform to the categorization in Fig. 1A (see the Discussion Limitations section for more details). Sonomicrometry recordings were corrected for outliers and level shifts and upsampled to 1000 Hz for plotting.

## 3. Results

Normalized long-axis measurements for the under-pennate and reverse pennate markers were strikingly different from normalized fascicle length (inline) measurements (Fig. 1). Reverse-pennate markers exhibited the more dramatic differences, and in many cases, changed distance not with just different amplitude, but even in the opposite direction to that of the fascicle length. Under-pennate markers exhibited distance changes closer to that of fascicle lengths, but even so, at times showed very little length change or even reversed length changes. Potential explanations for these differences are considered in the Discussion.

Though long-axis distances exhibited markedly different temporal profiles from fascicle lengths, we wished to determine whether some consistent *relationship* might still exist between the long-axis distances and the (inline) fascicle lengths in the worst-case scenario. As such, we selectively chose a worst-case illustration of divergence (Bird E), and we plotted fascicle lengths versus long-axis distances for the various muscle protocols (see Fig. 2, panels C, D). We found that the curves doubled back on themselves, preventing any attempts at confidently mapping from long-axis distance to fascicle length. Curve shapes also varied between protocols, particularly between isometric (second row of figure) and others, and between passive (third row) and active (fourth row) shortening ramps. In particular, observing the data from the fixed-end tetanus trial, we found that the data even exhibit a full loop, further demonstrating the absence of a direct functional relationship.

## 4. Discussion

Properly placing muscle markers is an essential step in obtaining reliable fascicle length measurements. Long-axis distance measurements can not only differ from fascicle length; they can even change in the opposite direction. Further, long-axis measurements can also bear no consistent relationship with fascicle length, suggesting that there is no reliable substitute to placing markers inline with the fascicle.

Upon finding differences *between* our long-axis distance measurements, we further investigated (in Turkeys C-E) the effect of marker pair orientation on these differences (though μCT-measured orientations did not consistently match our intended orientations, as discussed in Limitations below). Attempts at under-pennate marker placements resulted in smaller distance change measurements than the fascicle length changes, and attempts at reverse-pennate marker placements resulted in even smaller or opposite distance change measurements.

One possible explanation for this diverging distance change behavior can be seen using the historical parallelepiped muscle model used by Steno (Kardel, 1990), which is geometrically similar to the unipennate turkey lateral gastrocnemius (see Fig. 3A) and holds both the thickness of the muscle and the lengths of its aponeuroses fixed. The simplified two-dimensional version of this model – the parallelogram muscle model – is equivalent to a parallelogram under shear transformation (see Fig. 3B). This parallelogram muscle model predicts that under-pennate and reverse-pennate markers exhibit reduced and reversed distance changes, respectively, in comparison with fascicle length changes (see Fig. 3C), with strong similarities to our findings (for further predictions of this model, see Supplementary Fig. 1). Note that this model does not contradict the concept of an architectural gear ratio (Brainerd and Azizi, 2005) (see Supplementary Fig. 2).

While the parallelogram muscle model offers one possible explanation for seeing reverse-action measurements, it also *erroneously* predicts that muscle fascicle lengths can be predicted from markers arbitrarily placed along the long axis of the muscle. To the contrary, we found that long-axis measurements lacked a consistent relationship with fascicle lengths across the variety of muscle protocols, with curves doubling back on themselves and even forming loops. When a loop forms, the long-axis distance corresponds to two unique fascicle lengths, demonstrating that a one-to-one mapping does not exist. This lack of mapping from muscle long-axis distance to fascicle length is critical knowledge to anyone requiring fascicle length information: fascicle length information is not obtainable from long-axis distance length signals.

These results provide insight into the importance of furthering our scientific understanding of the complexity of muscles. The lack of mapping from long-axis distance lengths to fascicle lengths emphasizes that additional, distinct information exists outside that obtained by simply using pairs of markers. This highlights the value in developing new tools to track volumetric muscle deformation. For instance, the field of muscle physiology would strongly benefit from the further development and use of new tools or techniques employing marker constellation tracking (Aeles et al., 2023), three-dimensional ultrasound reconstruction (Sahrmann et al., 2024), and other approaches accounting for full muscle geometry (Orsbon et al., 2018). Such techniques will empower us to better explore and further understand neuromuscular diseases, fast and slow twitch muscle dynamics, intramuscular differences in fiber contractions, and other principles of muscle function. And while these results illuminate a large opportunity for growth, they also raise a strong caution. Critically, there are real pitfalls to placing markers incorrectly, with the potential consequences of developing incorrect models or collecting data that is not usable for a given application.

In addition to providing insight into scientific investigation, this work also has significant engineering implications. For example, if a marker pair strategy (such as magnetomicrometry (Taylor et al., 2021)) is used to control a prosthesis, when implanted within a pennate muscle, the markers must be implanted with sufficient alignment with the fascicles to ensure the output signal sufficiently corresponds to muscle contraction.

Where possible, we recommend placing the muscle markers in a fascicle that is clearly visible to the eye, as we did here. We specifically did not implant the inline markers under the aponeurosis, so that we would have a clear view of the location of the marker placement along a fascicle. Other strategies include the use of ultrasound guidance during insertion, though this may be prone to manual error, or the use of electrical stimulation on one side of the muscle and visual observation of dimpling on the other to track the fascicle path (Griffiths, 1987). In the absence of any of these strategies, in relevant species and muscles, the aponeuroses can be used as guides for the rough direction of the pennation angle to be matched by the marker orientation.

## 5. Limitations

Though we have discussed muscles here in the context of their three-dimensional complexity, we have limited our exploration, terminology, and modeling to two dimensions. A simple two-dimensional model roughly predicts some of the types of differences in measurements we observed, but the results of the model do not predict the muscle behavior in general, suggesting other models may better describe the geometry across the range of muscle activities, and further highlighting the need for robust real-time three-dimensional imaging tools. Further, we note that regional differences in contraction patterns likely contributed to our results, and that tools that can monitor heterogeneity in fascicle contractions are also needed.

We emphasize that the accuracy of our μCT imaging was substantially limited, with orientation measurements being highly sensitive to the replacement of tracking markers with imaging markers or to small tugs on the sonomicrometry wires during preparation of the specimens for imaging. The angles we show in Fig. 1B are rough approximations, and are given relative to the long-axis of the muscle, without knowledge of the actual pennation angle. However, despite the significant need for the development of new methods for validating marker positions, the conclusions derived from this work are clear: long-axis distance measurements are simply not a substitute for fascicle length when placed at shallow angles (regardless of whether they are under-pennate or reverse-pennate) into the muscle.

We also further emphasize that our results demonstrate what *can* happen with muscle long-axis distance measurements, not what *will* happen. We do not claim that correlation between fascicle length and long-axis measurements is not possible; we simply demonstrate that it is possible for fascicle length to be not predictable from long-axis measurements.

## 6. Conclusion

When placing muscle marker pairs for fascicle length measurement, whether via sonomicrometry, fluoromicrometry, magnetomicrometry, or any other marker-based tracking method, the markers must be implanted inline with the fascicle to provide reliable fascicle length change information. Errors in marker pair orientation result in differing, and sometimes even opposite, length change measurements. Particularly, long-axis measurements, whether below or opposite the pennation angle, provide signals sufficiently distinct that they bear no consistent relationship to the fascicle length. We thus strongly advocate for biomechanics researchers and surgeons to place muscle tissue length markers directly along muscle fascicles when investigating fascicle length or implementing clinical muscle tissue length tracking.

## Supplementary Material

1

## Figures and Tables

**Fig. 1.**
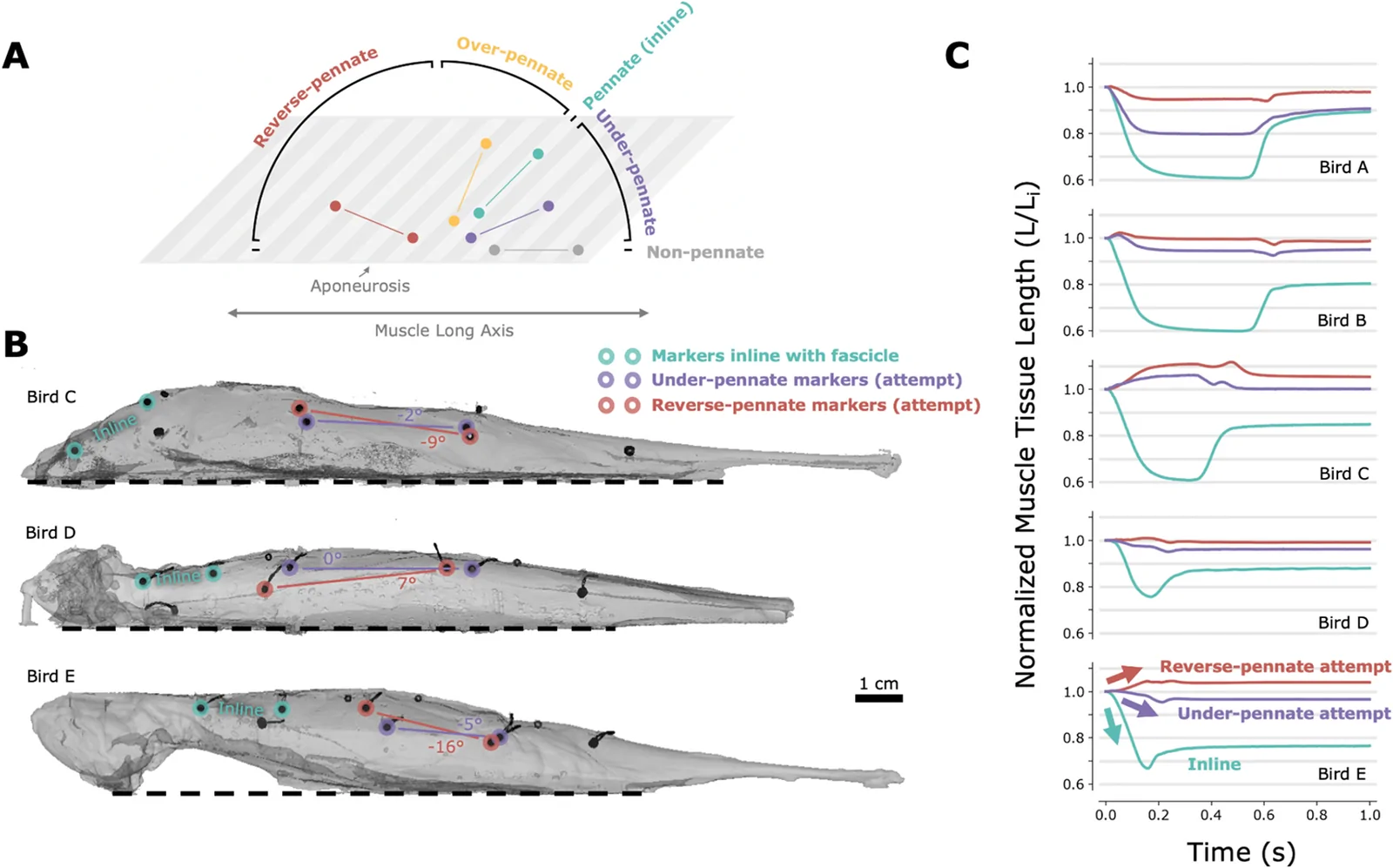
Inverted Length Change in Seemingly-Aligned Long-Axis Muscle Distance Markers. This figure demonstrates that markers aligned roughly along the long axis of a muscle from a superficial view can experience a change in distance opposite to that of the fascicle length change. **(A)** Diagram of different possibilities for marker pair orientations in relation to the pennation angle when implanted at different depths from the superficial muscle surface along the long-axis of the muscle. Only three of the five possible orientation classifications shown here are investigated in this work (pennate, under-pennate, and reverse-pennate). Pennate markers are oriented along the fascicle, while under-pennate and over-pennate muscles have smaller or larger angles to the long-axis, respectively. Reverse-pennate markers form an angle with the long axis in the opposite direction as the fascicles, and non-pennate markers are oriented directly along the long-axis of the muscle (parallel to the aponeurosis). The muscle diagram is theoretical and does not account for spatial variations in fascicle orientations throughout the muscle. **(B)** μCT images showing sonomicrometry transducers in the gastrocnemius muscles of Turkeys C, D, and E (lateral view, with the distal sides to the right of the images and the superficial surface at the top of each image). The skewed geometry of Turkey E is due to position during fixation with an underlying bone in place. We placed a pair of transducers (highlighted in teal) inline with the proximal fascicles in each turkey (where we could see the fascicle by eye during implantation), and then attempted to place a pair of transducers in an under-pennate (purple) and reverse-pennate (red) orientation along the long-axis of the muscle. Angles to the long-axis are noted in corresponding colors. While the angles do not fully conform to the categorization in panel A, recognizing the limitation in our μCT fixation and measurement methodology, we have left the markers highlighted by their *intended* orientations. Note also that, despite the skew in the proximal side of the fixed muscle causing the inline markers to appear with various orientations, the inline markers were visually confirmed to be placed along the fascicle during implantation. **(C)** Muscle tissue length versus time for each of the sonomicrometry pairs under isokinetic muscle shortening at 120 mm/s. Muscle tissue lengths are shown for each of the five turkeys A through E and are normalized according to their starting length (note that the starting length L_i_ used to normalize the muscle tissue lengths differs from the resting length L_0_ because the muscle starts stretched for the isokinetic shortening protocol). Note how the long-axis distances (plotted in purple and red) are not sufficient to predict the (inline) fascicle lengths (plotted in teal), sometimes even showing an inverted length change. Arrows in the bottom plot emphasize the diverging length changes between the long-axis lengths in turkey E.

**Fig. 2.**
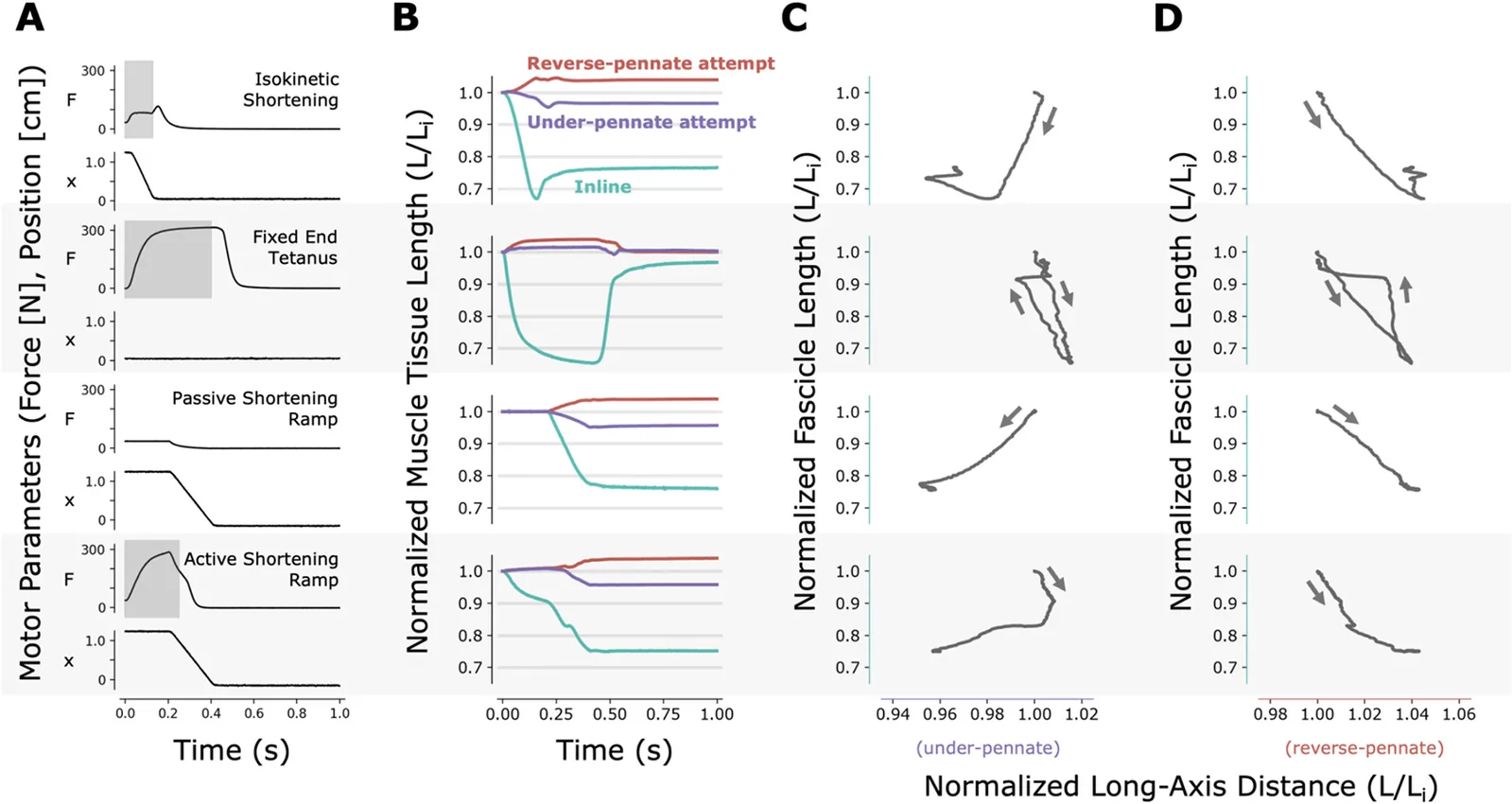
Lack of a consistent map from long-axis distances to fascicle lengths. Data from Turkey E showing the lack of a consistent map from long-axis distances to fascicle lengths. Note that the starting length L_i_ used to normalize the muscle tissue lengths differs from the resting length L_0_ in all but the fixed end tetanus case because the muscle starts stretched for the shortening protocols. **(A)** Motor parameters (force F and position x) shown for each of four muscle protocols (gray background highlighting in the force plots indicates the stimulation period). **(B)** Muscle tissue length versus time for each of the sonomicrometry pairs under the various muscle protocols. **(C, D)** Fascicle lengths (inline measurements) are plotted versus long-axis distances, with panel C showing fascicle lengths versus under-pennate marker distances and panel D showing fascicle lengths versus reverse-pennate marker distances. All data are normalized relative to initial distance. Arrows show the direction of the flow of time of the measurements. Note the loops in the Fixed End Tetanus plots (second row), preventing a reliable functional mapping from long-axis distances to fascicle lengths. Note also the inconsistent relationship between long-axis distances and fascicle length during shortening across the different muscle force protocols.

**Fig. 3.**
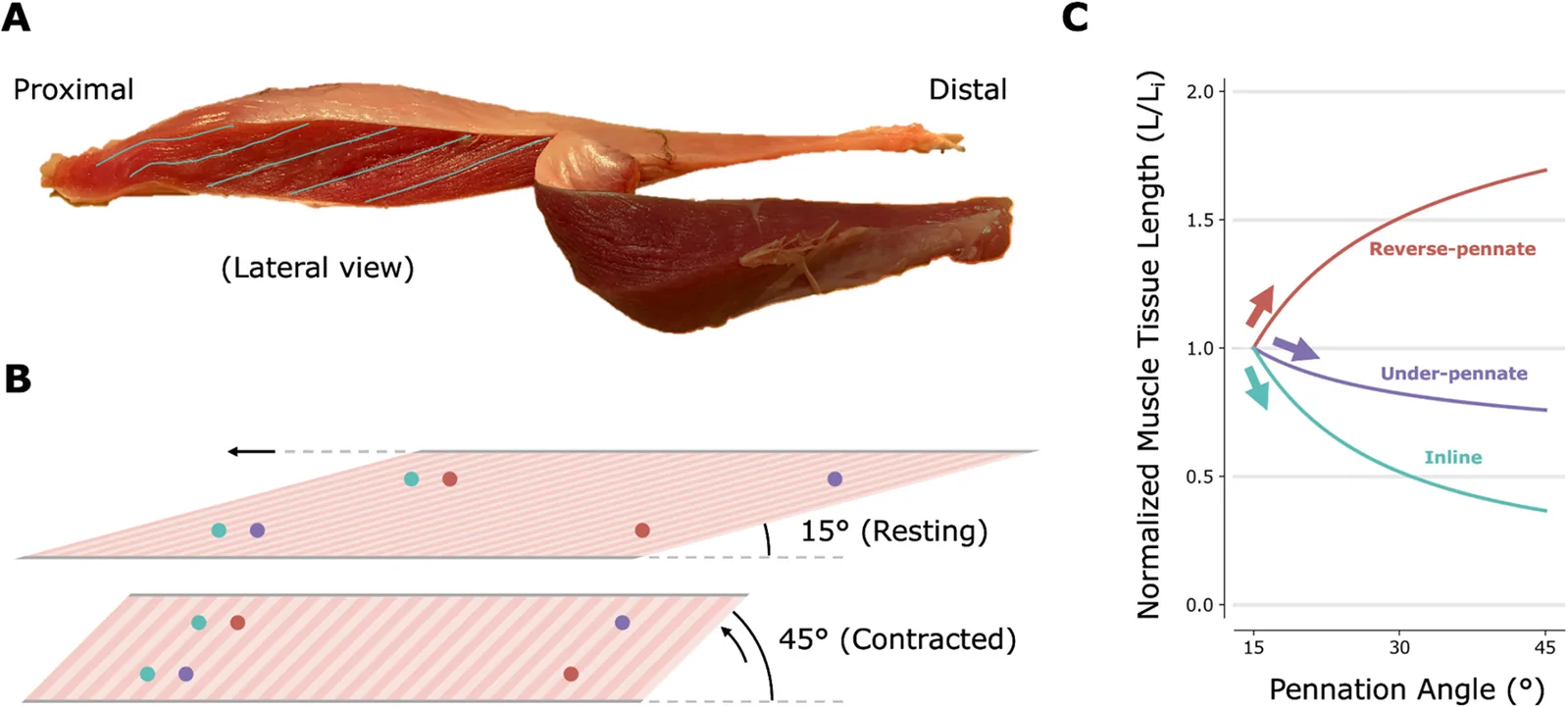
Possible Mechanism for the Inverted Length Change in Marker Distance. We investigated one possible mechanism for the reverse action of marker distances using a two-dimensional parallelogram muscle model, where the proximal (deep) aponeurosis is fixed in length and position and where the distal (superficial) aponeurosis is also fixed in length but is allowed to translate parallel to the proximal aponeurosis. **(A)** Photograph of dissected turkey gastrocnemius with fascicle paths (teal) traced by hand. **(B)** Diagram of the mathematical model of marker distances under contraction of the parallelogram muscle model, with inline markers (teal) oriented pairwise along the fascicle, under-pennate markers (purple), and reverse-pennate markers (red). Aponeuroses (gray) are fixed in both length and distance apart, so all motion is accomplished by combined shortening and rotation of the fascicles (shown in alternating shades of pink) within those constraints. **(C)** Distances between markers as the muscle contracts (normalized by initial distances) from a pennation angle of 15 degrees to a pennation angle of 45 degrees. While the distance between the inline markers decreases with increasing pennation angle, the reverse-pennate markers become further apart as the pennation angle increases. The under-pennate markers do not provide the same information as the fascicle length, but in this model they do exhibit the same sign in their length change, suggesting they may be a better option than a reverse-pennate orientation if inline implantation is not possible.

## Data Availability

All data needed to evaluate the conclusions in the paper are present in the paper and the supplementary materials.

## Appendix A. Supplementary data

Supplementary data to this article can be found online at https://doi.org/10.1016/j.jbiomech.2025.113137.
