# Supplementary material for: A critical orientation on muscle marker placement: Multiple angles to consider
Source: J Biomech. Author manuscript; Available in PMC 2026 Oct 27. (PMC13712260; doi:10.1016/j.jbiomech.2025.113137)
Supplement: Supplementary file 1 [file NIHMS2208372-supplement-1.docx]

**Supplementary Materials**

*Glossary*

**Pennate (inline) markers:** markers that are in line with their fascicle

**Under-pennate markers:** when the line between the markers has an angle that is between that of the long axis of the muscle and that of the muscle fascicles

**Reverse-pennate markers:** when the line between the markers has a slope that is opposite in sign to that of the muscle fascicles relative to the long axis of the muscle.

**Non-pennate makers:** when the line between the markers is parallel to the long axis of the muscle

**Over-pennate markers:** when the line between the markers has an angle that is greater than the pennation angle and has a same-signed slope as the muscle fascicles relative to the long-axis of the muscle (at this angle, the markers are in danger of becoming reverse-pennate during contraction).

*Nerve Stimulation for Muscle Contraction*

To stimulate muscle contraction, prior to placing the sonomicrometry transducers, we placed a custom-made nerve cuff on the tibial branch of the sciatic nerve, cut the nerve proximally, and bathed the area in mineral oil. Supramaximal 0.2 ms square wave pulses at 100 Hz were delivered using a Grass S48 stimulator.

*Muscle Overall Length Control and Muscle Force Measurement*

To measure muscle forces and control overall muscle length, we rigidly affixed the muscle origin (the femur) to a rigid aluminum frame, cut the fleshy end of the distal tendon, and attached it in series to a tension load cell (Omega Engineering Inc. LC703-500) and a linear servomotor (Kollmorgen AKM42G-EKCNC-00) by clamping the bony element in the tendon. Muscle force and overall muscle length were recorded via a National Instruments A-D converter (USB 6218). Servo motor position was controlled by Kollmorgan software.

*Micro CT Methods*

Lateral gastrocnemius muscles were removed for micro-CT scanning post-experimentation in three turkeys. For Turkey C, we replaced the sonomicrometry transducers with radiopaque beads prior to scanning in an effort to prevent artifacts that could obscure marker positions. We carefully attempted to insert these beads in the same place as the sonomicrometry transducers we had removed, following the same path and depth as the original transducers. We then scanned the muscle before rigor mortis. Subsequent muscles (Turkeys D and E) were extracted post-rigor with the transducers intact to maintain muscle shape and marker positions. During scanning, we wrapped each muscle in foam sheeting and sealed it in a plastic bag in an effort to minimize shifting and dehydration. Micro-CT scanning was performed using a Bruker Skyscan 1276 at Brown University (60 kV X-ray source, 41 μm resolution, 200 μA current, and a 0.5 mm aluminum filter). We constructed the image stacks using SkyScan NRecon software and segmented them in 3D Slicer (freeware, open source, https://www.slicer.org).

*Supplementary Figures*

**Supplementary Figure 1.** This figure shows how instantaneous normalized length change varies with marker pair orientation (angle relative to the long-axis of the muscle) in the context of a parallelogram muscle model (shear deformation - constant muscle thickness and parallel, constant-length aponeuroses) at the start of measurement (at which point the measurements are normalized). **(A)** This diagram shows a marker moving at constant (full muscle) velocity, v, relative to another, fixed, marker. The vertical distance between the markers (the muscle thickness, c) is fixed. The *starting* angle between the marker orientation and the long-axis of the muscle is given by θ and the distance between the markers is given by d. Equations for the parameters of interest are shown below the diagram. **(B)** The instantaneous distance change between the markers is shown as a function of the marker orientation as the top right marker moves at a constant velocity to the left. Background colors correspond to marker orientations as given in the legend in Figure 1A. Note that when θ is small the distance change approaches the marker velocity. **(C)** The distance between the markers shown as a function of marker orientation. **(D)** The instantaneous normalized distance change is found by dividing plot (B) by plot (C). Note that the *normalized* muscle distance changes less for under-pennate markers, and it changes with opposite sign for reverse-pennate markers. Over-pennate markers first have a greater, then a smaller, normalized instantaneous distance change depending on their orientation (and are in danger of becoming reverse-pennate markers if starting at a high angle). Unfortunately, modeling the markers affixed to opposing aponeuroses means that the distance between them approaches infinity for small θ, so panel (B) shows a distance change for non-pennate markers which is equal to the marker velocity, which is true for parallel-fibered muscles but not for pennate muscles under the parallelogram muscle model. However, dividing by the distance here compensates for this non-physical result, correctly showing that side-by-side (non-pennate) markers under shear transformation do not change the distance between them.

**Supplementary Figure 2.** This figure gives the same plot as given in Figure 3C, but without normalization and showing the difference in muscle length from its starting point (inline in teal, under-pennate in purple, and reverse-pennate in red). Brainerd and Azizi define architectural gear ratio (AGR) as longitudinal strain divided by fiber strain, and they predict that AGR should always be equal to or greater than 1, suggesting that the change in a non-normalized under-pennate measurement should be greater than the change in a non-normalized inline measurement. This is true here, with the non-normalized under-pennate marker distance (purple) decreasing in length more than the fascicle length (teal), and is true in general for this model (see Supplementary Figure 1B). This shows that this model does not contradict and perhaps even provides one possible contribution to the architectural gear ratio (AGR), though insufficient to account for the size of a typically measured AGR, as AGR is primarily a volume-mediated effect, and our simplistic two-dimensional model predicts only a small strain ratio.
